# Customized chemotherapy based on *epidermal growth factor receptor* mutation status for elderly patients with advanced non-small-cell lung cancer: a phase II trial

**DOI:** 10.1186/1471-2407-12-185

**Published:** 2012-05-21

**Authors:** Shiro Fujita, Nobuyuki Katakami, Katsuhiro Masago, Hiroshige Yoshioka, Keisuke Tomii, Toshihiko Kaneda, Masataka Hirabayashi, Kei Kunimasa, Toshio Morizane, Tadashi Mio

**Affiliations:** 1 Institute of Biomedical Research and Innovation Hospital, 2-2 Minatojima Minami-machi, Chuo-ku, Kobe, 650-0047, Japan; 2 Kyoto University Hospital, 54 Shogoin Kawaracho, Sakyo-ku, Kyoto, 606-8507, Japan; 3 Kurashiki Central Hospital, 1-1-1 Miwa, Kurashiki-shi, Okayama, 710-8602, Japan; 4 Kobe City Medical Center General Hospital, 2-1-1 Minatojima Minami-machi, Chuo-ku, Kobe, 650-0047, Japan; 5 Kobe City Medical Center West Hospital, 2-4 Ichibancho, Nagata-ku, Kobe, 653-0013, Japan; 6 Hyogo Prefectural Amagasaki Hospital, Higashidaimotsu-cho, Amagasaki, Hyogo, 660-0828, Japan; 7 Kanagawa Dental College, 82 Inaokacho, Yokosuka-ku, Kanagawa, 238-8580, Japan

## Abstract

**Background:**

Elderly patients are more vulnerable to toxicity from chemotherapy. Activating *epidermal growth factor receptor* (*EGFR*) mutations in non-small-cell lung cancer (NSCLC) are associated with enhanced response to EGFR tyrosine-kinase inhibitors. We studied patients with advanced NSCLC for whom treatment was customized based on *EGFR* mutation status.

**Methods:**

We screened 57 chemotherapy-naïve patients with histologically or cytologically confirmed NSCLC, stage IIIB or IV, aged 70 years or older, and with an Eastern Cooperative Oncology Group performance status 0 or 1, for *EGFR* exon 19 codon 746–750 deletion and exon 21 L858R mutation. Twenty-two patients with *EGFR* mutations received gefitinib; 32 patients without mutations received vinorelbine or gemcitabine. The primary endpoint was the response rate.

**Results:**

The response rate was 45.5% (95% confidence interval [CI]: 24.4%, 67.8%) in patients with *EGFR* mutations and 18.8% (95% CI: 7.2%, 36.4%) in patients without *EGFR* mutations. The median overall survival was 27.9 months (95%CI: 24.4 months, undeterminable months) in patients with *EGFR* mutations and 14.9 months (95%CI: 11.0 months, 22.4 months) in patients without *EGFR* mutations. In the gefitinib group, grade 3/4 hepatic dysfunction and dermatitis occurred in 23% and 5% of patients, respectively. In patients treated with vinorelbine or gemcitabine, the most common grade 3 or 4 adverse events were neutropenia (47%; four had febrile neutropenia), anemia (13%), and anorexia (9%). No treatment-related deaths occurred.

**Conclusions:**

Treatment customization based on *EGFR* mutation status deserves consideration, particularly for elderly patients who often cannot receive second-line chemotherapy due to poor organ function or comorbidities.

**Trial registration:**

This trial is registered at University hospital Medical Information Network-clinical trial registration (http://www.umin.ac.jp/ctr/index/htm) with the registration identification number C000000436.

## Background

In many developed countries, lung cancer is the leading cause of cancer death for both men and women. Approximately 80% to 85% of lung cancer subtypes are non-small-cell lung cancer (NSCLC). Like other solid tumors, NSCLC primarily occurs in the elderly and most patients are diagnosed with advanced disease that is unsuitable for surgery [1,2]. As the geriatric population inexorably increases, more elderly patients with NSCLC will receive anticancer agents for palliative intent. Because definite potential exists for higher toxicity, attributed to progressive organ failure and comorbidities, single-agent chemotherapy (using a third-generation agent) along with platinum-based combinations is recommended for the treatment of this population [3,4].

Theoretically, drug-induced adverse events (AEs) are avoidable while maintaining anti-tumor effects by targeting the critical molecule that drives proliferation of and is solely present in cancer cells. After the success of imatinib for treatment of BCR-ABL-dependent chronic myelogenous leukemia [5,6], understanding the molecular basis of human cancer has been exploited to provide targeted drugs. The epidermal growth factor receptor (EGFR) is a receptor tyrosine kinase (TK) of the ErbB family that has been implicated in cell proliferation and survival [7]. EGFR is a target of the tyrosine kinase inhibitor (TKI) gefitinib (Iressa; AstraZeneca, Macclesfield, United Kingdom), which has been approved for NSCLC treatment in many countries. The EGFR-TKIs achieved responses of approximately 10% in phase III clinical trials in patients with previously treated, unselected patients with advanced NSCLC [8,9]. Responses of EGFR-TKIs were more likely among certain patients:women, never- or light-smokers, patients with adenocarcinoma, and Asians [10-12]. In 2004, somatic mutations in the kinase domain of *EGFR* were predominantly found in these patients and were linked to EGFR-TKI sensitivity [13-15]. More than 90% of these mutations are observed in two hotspots: in-frame deletions including amino acids at codons 747 to 749 in exon 19, and an amino acid substitution at codon 858 (L858R) in exon 21 [16-18]. These mutations are postulated to mediate oncogenic effects by altering downstream signaling and anti-apoptotic mechanisms [13-15]. Prior trials confirmed that the response rate to EGFR-TKI in NSCLC patients with *EGFR* mutations is approximately 70–80% [12,19,20], and results of recent phase III trials showed that the oral EGFR-TKI gefitinib has a superior progression-free survival (PFS) to standard chemotherapy as the first-line therapy for NSCLC with mutated *EGFR*[21,22].

Mutations are found frequently in individuals of East-Asian ethnicity (30%), and there are expected clinical benefits to gefitinib treatment in these patients [23]. For elderly patients with advanced NSCLC and wild-type *EGFR*, single-agent chemotherapy with a third-generation agent can be considered to be a recommended option. To maximize the effect of treatment, customized treatment based on *EGFR* mutation status would be mandatory. To evaluate the clinical feasibility and efficacy of such customized therapies, we conducted a prospective clinical trial that included patient assignment based on *EGFR* mutation status.

## Methods

### Eligibility

The study was a phase II, open-label, non-randomized, multicenter study (a phase II study of Iressa versus Vinorelbine or gemcitAbine in chemo-naïve elderly patients with advanced Non-small-cell lung cancer based on epidermal growth factor receptor mutation status: IVAN Trial; University hospital Medical Information Network-clinical trial registration number, C000000436). Patients enrolled in the present study had measurable, pathologically confirmed stage IIIB or IV NSCLC and were aged 70 years or older. Availability of archived tumor tissue or pleural/pericardial fluid for evaluation of *EGFR* mutation status was required for enrollment. Other eligibility criteria included written informed consent; no prior chemotherapy; Eastern Cooperative Oncology Group (ECOG) performance status (PS) 0 or 1; adequate bone marrow, renal, and hepatic function; and a life expectancy of at least 3 months. Exclusion criteria included symptomatic brain metastasis, any evidence of interstitial lung disease on chest computed tomography examination, other co-existing malignancies or malignancies diagnosed within the last 5 years other than carcinoma *in situ*, history of congestive heart failure, unstable angina pectoris or recent history (within 6 months) of acute myocardial infarction, uncontrolled cardiac arrhythmia, severe psychiatric illness, or concurrent disease or condition that would have made the patient inappropriate for study participation. The local ethical committees (the Research Ethics Committee of the Institute of Biomedical Research and Innovation, the Ethics Committee of Kyoto University Graduate School and Faculty of Medicine, the Clinical Research Approval Committee and the Medical Ethics Committee of Kurashiki Central Hospital, the Ethics Committee for Clinical Research of Kobe City Medical Center General Hospital, the Research Ethics Committee of Kobe City Medical Center West Hospital, and the institutional review board at the Hyogo Prefectural Amagasaki Hospital) approved the study, and written informed consent was obtained from each patient. The study was conducted in accordance with the Helsinki declaration.

### EGFR mutation screening

In the current open-label study, treatment was assigned based on *EGFR* mutation status. First, the *EGFR* gene mutational status of a biopsy specimen from each patient was evaluated. Initially, patients were screened for *EGFR* mutations in a commercial central laboratory at SRL in Tokyo. Both the exon 19 deletion mutation and the L858R point mutation were screened by direct sequencing, as described previously [13]. The T790M mutation, which was reported to be associated with resistance to EGFR-TKI therapy, was also checked, and the patient was excluded from the study if this mutation was detected [24]. From June 1, 2007, outsourcing of EGFR genetic testing was covered by government insurance in Japan, and the protocol was amended to allow the outsourcing from each institution to commercial clinical laboratories, either at Mitsubishi Chemical Medience in Tokyo (peptide nucleic acid-locked nucleic acid PCR clamp method) [25] or BML, Inc. in Tokyo (PCR-invader assay) [26]. During the study period, the methodology for *EGFR* mutation detection was substantially improved, and direct sequencing was substituted with highly-sensitive, advanced-generation detecting technology available in Japan.

### Treatment plan

Patients whose tumor contained an *EGFR* activating mutation received gefitinib (250 mg per day orally). In the present study, gefitinib was selected as the EGFR-TKI. Gefitinib was first approved in Japan for treatment of patients with advanced NSCLC on July 5th, 2002, and Japanese oncologists had already acquired considerable experience with this drug at the beginning of the present study. Patients with no *EGFR* activating mutation were treated with cytotoxic chemotherapy: either vinorelbine 25 mg/m^2^ on Day 1 and 8 (the recommended dose in Japan) or gemcitabine 1000 mg/m^2^ on Day 1 and 8. The choice of the chemotherapy regimen was at the discretion of the attending physician, and a total of three to six cycles of treatment were required for either treatment regimen. Treatment was continued until progression of the disease (PD), development of unacceptable toxicity, patients’ request, or completion of the treatment. Further therapy after PD was permitted in the protocol.

Dose modification of gefitinib, vinorelbine, or gemcitabine was allowed according to the protocol. The gefitinib dosing schedule could be modified to every second day for patients with severe toxicity: pulmonary toxicity (except for cough, forced expiratory volume in 1 second, hiccoughs, interstitial pneumonitis/pulmonary infiltrates) ≥ grade 2, or grade 3/4 diarrhea, mucositis, liver dysfunction, hematologic toxicity, skin toxicity, or ocular toxicity. For vinorelbine, a maximum of one dose reduction (25 to 20 mg/m^2^) was allowed based on the following criteria: neutropenic fever, ileus ≥ grade 2, non-hematological toxicity (except for nausea, hyponatremia, body weight loss, anorexia, and alopecia) ≥ grade 3, grade 4 neutropenia and/or thrombocytopenia. Regarding gemcitabine, a maximum of one dose reduction (1000 to 800 mg/m^2^) was allowed based on the following criteria: neutropenic fever, interstitial pneumonitis or pulmonary fibrosis ≥ grade 1, non-hematological toxicity (except for nausea, hyponatremia, body weight loss, anorexia, and alopecia) ≥ grade 3, grade 4 neutropenia and/or thrombocytopenia.

The primary outcome of the study was the objective response rate (ORR): partial plus complete response based on the Response Evaluation Criteria in Solid Tumors [27]. The present study was designed to detect a response rate of 65% in the gefitinib group compared with a minimal, clinically meaningful response rate of 30%. It was estimated before starting the present study that 20 eligible patients would allow the study to have 80% power to detect this difference, with an alpha level of 0.05. The drop rate was considered to be 10%, and a total of 22 patients would need to be enrolled in the gefitinib group. We estimated that the frequency of NSCLC with *EGFR* mutations accounted for 25% of the total NSCLC population, based on the results of the pre-protocol survey regarding the number of newly referred advanced NSCLC patients to two hospitals (Kyoto University Hospital and the Institute of Biomedical Research and Innovation Hospital; data not shown). To enroll 22 patients in the gefitinib group, 88 patients would need to be screened, and 66 of the 88 patients were expected to have tumors with wild-type *EGFR*. To detect a response rate of 20% compared with a minimal, clinically meaningful response rate of 5% in the single-agent chemotherapy group, 27 patients were required to maintain more than 80% power to detect this difference, with an alpha level of 0.05. Sixty-six patients, estimated in the pre-protocol period, were sufficient. We expected that patients who had predictive factors for containing an *EGFR* mutation (*i.e.* female, East-Asian origin, adenocarcinoma, and no history of smoking) were more likely to be recruited in our trial. In other words, patients with wild-type *EGFR* would be less likely to be enrolled. In this case, 27 patients were minimally required to detect the difference in the single-agent chemotherapy group. The drop rate was considered to be 10%, and a total of 30 patients would need to be enrolled in the single-agent chemotherapy group.

Planned secondary outcomes included the disease control rate, 1-year survival rate, overall survival (OS), time to treatment failure (TTF), and toxicity. Disease control was defined as the best response out of complete response, partial response, or stable disease, which was confirmed and sustained for 4 weeks or longer. TTF was measured from the start date of treatment to the date of discontinuation of the study treatment, occurrence of PD, or death by any cause (whichever occurred earlier). If intolerable toxicity or discontinuation of treatment secondary to toxicity occurred, the patient was considered assessable but was classified as a treatment failure.

During treatment, assessments were performed every 6 weeks until disease progression. Events were confirmed twice via source-document verification at site visits or delivered radiographic data by members of the data center and the investigators.

Safety was monitored by clinical AEs, laboratory (hematology and clinical chemistry) testing, radiographic information, and collection of vital signs, weight, and ECOG PS status. AE severity was graded based on the National Cancer Institute Common Toxicity Criteria (version 3.0). Interstitial pneumonitis was the most serious concern related to gefitinib treatment because this event has been noted in patients taking gefitinib.

OS and TTF were estimated by the Kaplan–Meier method, and the therapy arms were compared by log-rank test. Categorical variables were analyzed by Fisher’s exact test or *Χ*^2^ test. Continuous variables were assessed with unpaired *t*-tests or the Mann–Whitney *U*-test. Statistical analyses were performed using JMP version 6 (SAS Institute, Cary, NC, USA) and R software version 2.8.1 (R Foundation for Statistical Computing, Vienna, Austria).

## Results

### Accrual

Enrollment started in July 2006. From June 2007, the rate of enrollment slowed, mainly because government insurance coverage of *EGFR* genetic analysis had begun in Japan, and the benefit of enrollment in the present study was substantially lessened. The last patient was enrolled in April 2009. The data cut-off date used for the present report was February 10, 2011.

### Patients

**Figure 1:**
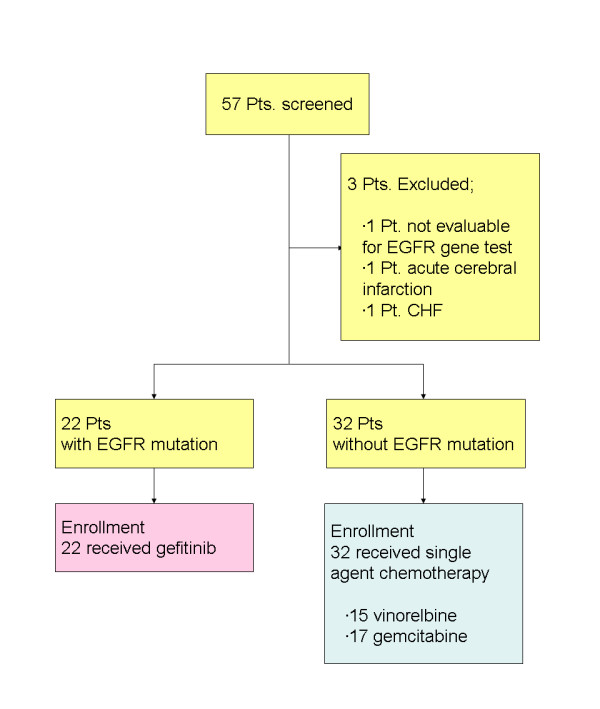
Study flow.

Fifty-seven patients were screened from July 2006 to April 2009 (Figure 1). Mutational analysis for one patient was unsuccessful because the amount of the specimen was insufficient. During analyses, two patients suffered serious illnesses not related to their cancer (acute atherothrombotic ischemic stroke of the right cerebrum in one patient and congestive heart failure due to constrictive pericarditis in another patient). These patients were deemed ineligible. Of the remaining 54 patients, 22 patients (41%) harbored mutations and 32 patients’ tumors were wild type. There was no patient whose tumor revealed the T790M mutation in exon 20, and all 54 patients were enrolled in the study. The demographic information of the patients is summarized in Table 1. As expected, some imbalances were observed between the treatment groups; more never-smokers (73% vs. 41%) and patients with adenocarcinoma histology (95% vs. 50%) were in the gefitinib group. These imbalances seemed to reflect the epidemiologic features of NSCLC harboring activating *EGFR* mutations. At the data cut-off, the median follow up was 15.8 months.

**Table 1:** Patient characteristics

	**All**	**Gefitinib**	**VNR or GEM**	***P***
Age (years)				
Median (range)	80 (71–89)	81 (71–85)	79 (72–89)	0.11
Male:Female	20:34	5:17	15:17	0.09
ECOG PS				
0:1	12:42	8:14	4:28	0.05
Histology				
Adeno	37	21	16	< 0.001
Squamous	8	1	7	
Other	9	0	9	
Smoking history				
Never	29	16	13	0.03
Former / current	25	6	19	
Mutation type				
Exon 19 del	6	6	―	―
Exon 21 L858R	14	14	―	
Ex19del + L858R	2	2	―	
Clinical stage				
IIIB	20	9	11	0.78
IV	34	13	21	

ECOG PS: Eastern Cooperative Oncology Group Performance Status; GEM: gemcitabine; VNR: vinorelbine.

### Response and survival of the gefitinib group

**Figure 2:**
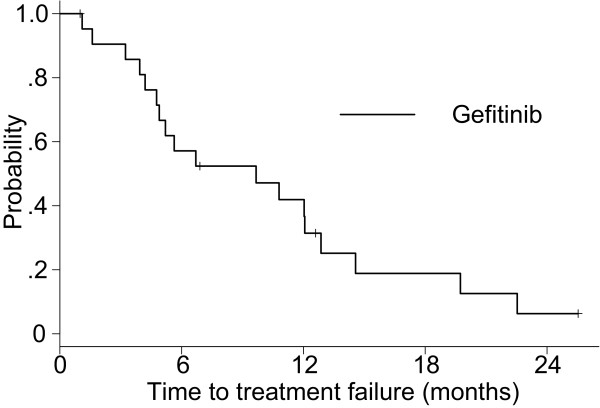
Time to treatment failure of the gefitinib group.

**Figure 3:**
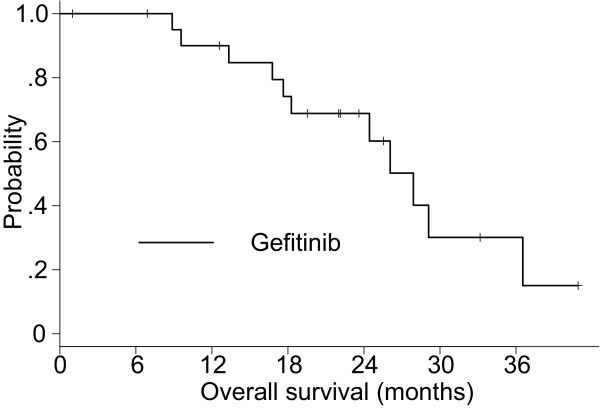
Overall survival of the gefitinib group.

A summary of the tumor response is shown in Table 2. The ORR was 45.5% (95% confidence interval [CI], 24.4–67.8%) and disease control rate was 86.4% (95% CI, 65.1–97.1%) for the gefitinib group. Eleven patients had died among the gefitinib group. The Kaplan–Meier estimate of the median TTF was 9.7 months (Figure 2) and the median survival time (MST) was 27.9 months (Figure 3). The 1-year OS rate was 90.0%.

**Table 2:** Best response according to RECIST in both groups

	**Gefitinib (N = 22)**	**VNR or GEM (N = 32)**
Complete response	0	0
Partial response	10 (45.5%)	6 (18.8%)
Stable disease	9 (40.9%)	12 (37.5%)
Progressive disease	1 (4.5%)	6 (18.8%)
Not evaluable	2 (9.1%)	8 (25.0%)

GEM: gemcitabine; RECIST: Response Evaluation Criteria in Solid Tumors; VNR: vinorelbine.

### Response and survival of the vinorelbine/gemcitabine group

**Figure 4:**
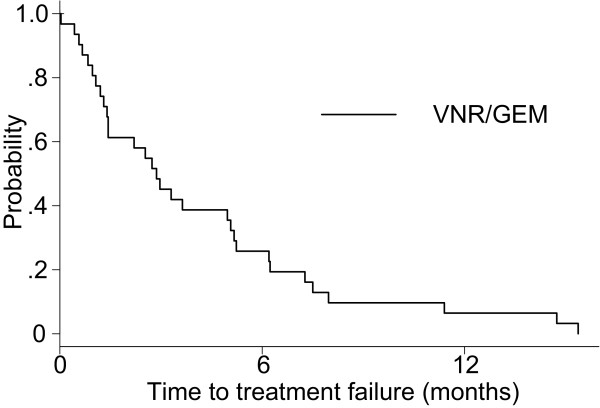
Time to treatment failure of the chemotherapy group.

**Figure 5:**
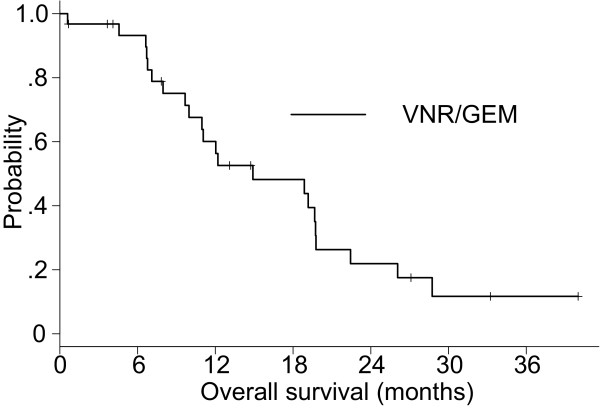
Overall survival of the chemotherapy group.

As shown in Table 2, the ORR was 18.8% (95% CI, 7.2–36.4%) and the disease control rate was 56.3% (95% CI, 37.7–73.6%) for the vinorelbine (VNR)/gemcitabine (GEM) group. Twenty-two patients had died among the VNR/GEM group. The Kaplan–Meier estimate of the median TTF was 2.9 months (Figure 4) and MST was 14.9 months (Figure 5). The 1-year OS rate was 60.1%.

### Comparison with regard to efficacy between the two groups

**Figure 6:**
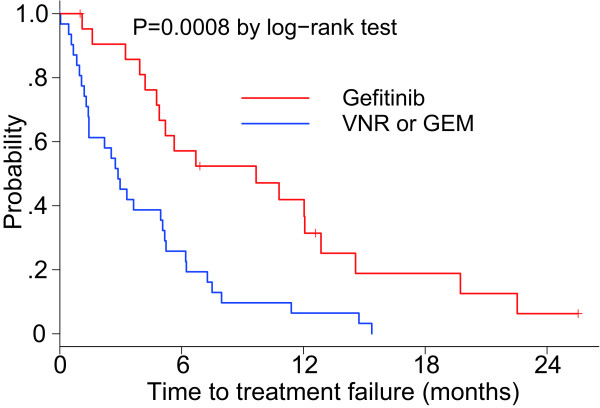
Kaplan–Meier curve of TTF according to the treatment group.

**Figure 7:**
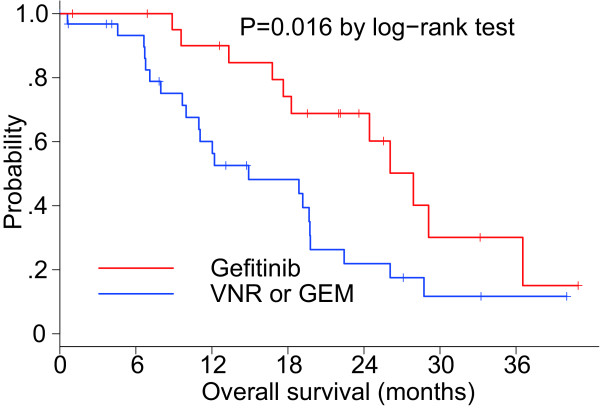
Kaplan–Meier curve of OS according to the treatment group.

**Figure 8:**
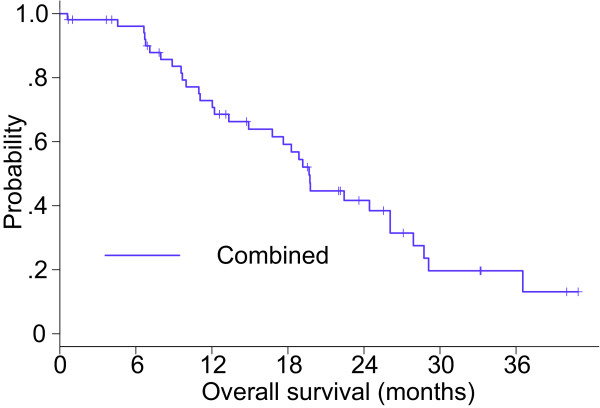
Kaplan–Meier curve of OS with all trial patients combined.

The baseline characteristics were different between the gefitinib and VNR/GEM groups because of the non-randomized nature of the study; however, a statistically significant difference was found regarding the disease control rate (86.4% vs. 56.3%; *P* = 0.035), but not regarding the ORR (45.5% vs. 18.8%; *P* = 0.067). Concerning the TTF, a statistically significant difference was observed between the groups (log-rank test; *P* = 0.0008; Figure 6). Additionally, a significantly longer OS was noted in the gefitinib group compared with the VNR/GEM group (log-rank test; *P* = 0.016; Figure 7), although there was a distinct difference between tumors in each group with regard to the *EGFR* mutation status. For all patients, the MST was 19.7 months (Figure 8).

### Second-line therapy

Of the 22 patients assigned to gefitinib, 14 received chemotherapy, 5 received radiotherapy, and 8 received EGFR-TKI (six received erlotinib, two underwent re-challenge with gefitinib); 2 received no therapy post-study. Of the 32 patients assigned to single-agent chemotherapy, 14 received second-line chemotherapy, 8 received radiotherapy, and 15 patients received EGFR-TKI (nine received erlotinib and six received gefitinib); five patients received no therapy post-study. No patient in the single-agent chemotherapy group was in the protocol-treatment phase.

### Toxicity

Toxicity information was available for all 54 treated patients. Treatment-related AEs were observed in 52 of the 54 treated patients. No treatment-related deaths occured in the gefitinib or VNR/GEM groups. AEs occurring in more than 10% of either treatment group are listed in Table 3. In the gefitinib group, skin-related AEs were observed in 14 of 22 patients (64%), and the most frequently occurring grade 3 or worse AE was elevation of aminotransferase. No interstitial lung disease was observed. Eleven patients (52%) in the gefitinib group had at least one drug discontinuation due to AEs; elevation of aminotransferases in six patients and skin toxicity in five patients.

**Table 3:** Adverse events (AEs) occurring in more than 10% of either treatment group

**Gefitinib (N = 22)**	**All Grades**	**CTC Grade 3 or 4**
Skin-related AEs	14 (63.6%)	1 (4.5%)
ALT	14 (63.6%)	5 (22.7%)
AST	12 (54.5%)	3 (13.6%)
Anemia	11 (50.0%)	0
Hypoalbminemia	9 (40.9%)	0
Anorexia	6 (27.3%)	0
Fatigue	6 (27.3%)	0
Diarrhea	6 (27.3%)	0
ALP	6 (27.3%)	0
**VNR or GEM (N = 32)**	**All Grades**	**CTC Grade 3 or 4**
Anemia	23 (71.9%)	4 (12.5%)
Leukocytopenia	21 (65.6%)	15 (46.9%)
Neutropenia	20 (62.5%)	15 (46.9%)
Fatigue	18 (56.3%)	2 (6.3%)
Anorexia	12 (37.5%)	3 (9.4%)
Thrombocytopenia	12 (37.5%)	1 (3.1%)
ALT	12 (37.5%)	0
Nausea	11 (34.4%)	2 (6.3%)
Constipation	9 (28.1%)	1 (3.1%)
AST	9 (28.1%)	0
Hyponatremia	5 (15.6%)	2 (6.3%)
Hyperkalemia	5 (15.6%)	0
Hypocalcemia	5 (15.6%)	0
Febrile neutropenia	4 (12.5%)	4 (12.5%)

AE: adverse event; ALT: alanine aminotransferase; ALP: alkaline phosphatase; AST: aspartate aminotransferase; CTC: common toxicity criteria; GEM: gemcitabine; VNR: vinorelbine.

As expected, hematologic AEs were common in the VNR/GEM group (Table 3). Grade 3 or 4 neutropenia, anemia, and thrombocytopenia occurred in 15 patients (47%), four patients (13%), and one patient (3%), respectively. Febrile neutropenia was observed in four patients (13%). The most common grade 3 or 4 non-hematologic AE in the VNR/GEM group was anorexia followed by fatigue, nausea, and hyponatremia. Dose reduction or treatment delay was documented in 14 patients (44%) due to neutropenia or neutropenic fever (eight patients), fever (three patients), patients’ request (two patients), or grade 3 constipation (one patient).

## Discussion

In the present study, the median TTF and MST of patients with mutated *EGFR* (treated with gefitinib) were 9.7 months and 27.9 months, respectively, which were comparable to the results of two recent trials. The North East Japan 002 Gefitinib Study Group conducted a phase III trial comparing an *EGFR* mutation-positive group of patients receiving gefitinib with carboplatin plus paclitaxel [21]. In the planned interim analysis, the PFS was significantly longer in the gefitinib group and the study was terminated. The gefitinib group had a significantly longer PFS compared with the chemotherapy group (10.8 months vs. 5.4 months, respectively; hazard ratio, 0.30; *P* <0.001). The MST for the gefitinib group was 30.5 months. Mitsudomi *et al.*, from the West Japan Thoracic Oncology Group, compared gefitinib with cisplatin plus docetaxel as the first-line treatment for advanced NSCLC with *EGFR* mutations [22]. The gefitinib group had a significantly longer PFS compared with the cisplatin plus docetaxel group, with a median PFS of 9.2 months vs. 6.3 months, respectively (hazard ratio, 0.489; *P* <0.0001). These results represent a milestone toward “tailor-made” therapy for NSCLC and gefitinib has been recently registered as the first-line treatment for NSCLC patients with *EGFR* activating mutations in Europe. Elderly patients have less of a chance of receiving second-line chemotherapy compared with their younger counterparts, due to poor organ function and/or comorbidities [28], and many elderly patients with relapsed or refractory NSCLC after first-line chemotherapy are deemed unfit for second-line chemotherapy [29]. Upfront administration of the most effective regimen (i.e., a regimen that is expected to have the longest PFS) is needed for these patients. The use of first-line gefitinib is valuable for elderly patients with *EGFR* mutant NSCLC.

No significant unexpected toxicity was found in the gefitinib group. Similar to other EGFR-targeted agents, skin-related AEs were the most common toxicity observed. No interstitial lung disease was reported. The Grade 3 to 4 elevation of alanine aminotransferase (ALT) and aspartate aminotransferase (AST) was observed in 22.7% and 13.6% of patients who received gefitinib, respectively. This result was comparable to previous studies conducted in Japan. The most common toxicity criteria (CTC)-AE grade 3 or 4 toxicity reported in the gefitinib group of the North East Japan 002 trial was aminotransferase elevation (26.3%) [21]. In the West Japan Thoracic Oncology Group 3405 trial, 14 of 87 patients (16.1%) experienced CTC-AE grade 3 or 4 AST elevation, and grade 3 or 4 ALT elevation was observed in 24 of 87 patients (27.6%) [22]. This liver enzyme elevation is almost always reversible; however, it may cause a decrease in dose intensity and may lessen efficacy.

The ORR in the gefitinib group was 45%, which seemed to be low compared with that reported in the literature [12,19,20]. One cause may be the high drug-discontinuation rate due to AEs. In the gefitinib group, 11 patients (50%) had drug discontinuation and dose intensity was lessened, which explains the low ORR in the gefitinib group. In the present study, all participants were aged 70 years or older, and the median age of the enrolled patients was 80 in the entire treatment population. Age-related decreases in organ function have the potential to increase drug-related toxicity in the elderly, although chronological age itself should not preclude appropriate treatment. Given the favorable TTF and OS in the gefitinib group, gefitinib was just as effective in the elderly with mutated *EGFR* compared with their younger counterparts. A comprehensive guide on dose adjustments in this population is mandatory, even for this recently developed, molecular-targeted agent.

Monotherapy with vinorelbine, gemcitabine, or docetaxel is still reasonable for patients with NSCLC of unknown *EGFR* mutation status (*i.e.*, both *EGFR* mutation-positive and -negative groups combined). In the key phase III study ELVIS (the Elderly Lung Cancer Vinorelbine Italian Study) [30,31], 161 chemotherapy-naïve patients (≥ 70 years old) were randomized to receive vinorelbine or best supportive care. The ORR was 20% and 1-year survival rate was 32% for patients receiving vinorelbine, and a significant survival benefit was recorded compared with the control group. In another key phase III study, the Multicenter Italian Lung Cancer in the Elderly Study, 700 patients were randomized to receive single-agent chemotherapy with vinorelbine or gemcitabine, or combination therapy with vinorelbine plus gemcitabine [32]. Combination treatment had no advantage regarding ORR, time to progression (TTP), or survival over single-agent therapy. The ORR was 18%, 16%, and 21% for vinorelbine, gemcitabine, and vinorelbine plus gemcitabine, respectively; the median TTP was 4.5, 4.3, and 4.8 months and MST was 9.0, 7.0, and 7.5 months, respectively. A phase III trial (West Japan Thoracic Oncology Group 9904), which compared docetaxel with vinorelbine for the treatment of elderly patients with advanced NSCLC, demonstrated a statistically significant improvement in median PFS (5.5 months vs. 3.1 months, respectively) and ORR (22.7% vs. 9.9%, respectively; *P* = 0.019), but no statistical difference was found regarding the MST (14.3 months vs. 9.9 months, respectively; *P* = 0.138) [29]. Recently, the IFCT-0501 phase III study demonstrated an improved OS of a platinum-based combination regimen (carboplatin plus paclitaxel) compared with single-agent chemotherapy; however, toxicity was an issue, as 9 of 143 (6.3%) patients died due to treatment-related AEs [33]. While the survival benefit with doublet chemotherapy is impressive, accompanying toxicity is a concern with this regimen.

In our study, single-agent chemotherapy was effective and feasible for the elderly patients with NSCLC who were wild type for *EGFR*. The ORR of 18.8% and MST of 14.9 months observed in the single agent chemotherapy group were compatible to the results of the previous clinical trials mentioned above. The major AEs were related to myelosuppression, were clinically manageable, and included no treatment-related deaths. Given the favorable results, we suggest that cytotoxic agent monotherapy should be considered to be the treatment for elderly patients with NSCLC who are wild type for *EGFR*.

At first, we planned a subsequent phase III study to evaluate the customized treatment based on *EGFR* mutation status, with patients randomized to either a control (conventional cytotoxic chemotherapy) arm or experimental (customized treatment) arm. In June 2007, however, outsourcing of *EGFR* genetic testing was fully covered by the health-insurance system in Japan, and customized treatment based on *EGFR* mutation status is a part of the practice for the treatment of NSCLC. Advanced-generation analytical methods, including highly-specific, rapid PCR techniques, allow us to detect *EGFR* mutations from fluid specimens, such as malignant effusion or aspiration-needle fluid, with satisfactory sensitivity and specificity. Most of patients with NSCLC have their tumor analyzed when the pathological diagnosis is confirmed, and the results of *EGFR* mutational analysis are usually available at the beginning of the first-line chemotherapy. Results of the present study represent outcomes of the current standard therapy for elderly patients with NSCLC in Japan, although the number of enrolled patients was small.

## Conclusions

This is the first prospective multi-center study in the elderly population with advanced NSCLC that included customized treatment based on *EGFR* mutation status. The MST of all participants (both groups combined) was 19.7 months, which was favorable in elderly patients (aged 70 years or older) with advanced NSCLC.

## Competing interests

The authors declare that they have no competing or financial interests.

## Authors’ contributions

SF and T. Mio designed the study. SF, NK, KM, HY, KT, TK, and MH contributed with provision of study materials or patients. SF, NK, KM, HY, KK, KT, TK, MH, and T. Mio contributed with collection and assembly of data. SF, NK, and T. Morizane performed statistical data analysis and helped with data interpretation. SF, NK, KM, HY, KK, KT, TK, MH, and T. Mio drafted the manuscript. All authors read and approved the final manuscript.

## Pre-publication history

The pre-publication history for this paper can be accessed here:

http://www.biomedcentral.com/1471-2407/12/185/prepub

## References

[B1] Edwards BK Howe HL Ries LA Thun MJ Rosenberg HM Yancik R Wingo PA Jemal A Feigal EG Annual report to the nation on the status of cancer, 1973–1999, featuring implications of age and aging on U.S. cancer burden Cancer 2002 94 10 2766 2792 10.1002/cncr.10593 12173348

[B2] Owonikoko TK Ragin CC Belani CP Oton AB Gooding WE Taioli E Ramalingam SS Lung cancer in elderly patients: an analysis of the surveillance, epidemiology, and end results database J Clin Oncol 2007 25 35 5570 5577 10.1200/JCO.2007.12.5435 18065729

[B3] NCCN Clinical Practice Guidelines in Oncology (NCCN Guidelines) Version 1.2011 2010

[B4] D'Addario G Fruh M Reck M Baumann P Klepetko W Felip E Metastatic non-small-cell lung cancer: ESMO Clinical Practice Guidelines for diagnosis, treatment and follow-up Ann Oncol 2010 21 Suppl 5 v116 v119 20555059 10.1093/annonc/mdq189

[B5] O'Brien SG Guilhot F Larson RA Gathmann I Baccarani M Cervantes F Cornelissen JJ Fischer T Hochhaus A Hughes T Imatinib compared with interferon and low-dose cytarabine for newly diagnosed chronic-phase chronic myeloid leukemia N Engl J Med 2003 348 11 994 1004 10.1056/NEJMoa022457 12637609

[B6] Druker BJ Guilhot F O'Brien SG Gathmann I Kantarjian H Gattermann N Deininger MW Silver RT Goldman JM Stone RM Five-year follow-up of patients receiving imatinib for chronic myeloid leukemia N Engl J Med 2006 355 23 2408 2417 10.1056/NEJMoa062867 17151364

[B7] Mendelsohn J Blockade of receptors for growth factors: an anticancer therapy–the fourth annual Joseph H Burchenal American Association of Cancer Research Clinical Research Award Lecture Clin Cancer Res 2000 6 3 747 753 10741693

[B8] Shepherd FA Rodrigues Pereira J Ciuleanu T Tan EH Hirsh V Thongprasert S Campos D Maoleekoonpiroj S Smylie M Martins R Erlotinib in previously treated non-small-cell lung cancer N Engl J Med 2005 353 2 123 132 10.1056/NEJMoa050753 16014882

[B9] Thatcher N Chang A Parikh P Rodrigues Pereira J Ciuleanu T von Pawel J Thongprasert S Tan EH Pemberton K Archer V Gefitinib plus best supportive care in previously treated patients with refractory advanced non-small-cell lung cancer: results from a randomised, placebo-controlled, multicentre study (Iressa Survival Evaluation in Lung Cancer) Lancet 2005 366 9496 1527 1537 10.1016/S0140-6736(05)67625-8 16257339

[B10] Fukuoka M Yano S Giaccone G Tamura T Nakagawa K Douillard JY Nishiwaki Y Vansteenkiste J Kudoh S Rischin D Multi-institutional randomized phase II trial of gefitinib for previously treated patients with advanced non-small-cell lung cancer (The IDEAL 1 Trial) [corrected] J Clin Oncol 2003 21 12 2237 2246 10.1200/JCO.2003.10.038 12748244

[B11] Kris MG Natale RB Herbst RS Lynch TJ Prager D Belani CP Schiller JH Kelly K Spiridonidis H Sandler A Efficacy of gefitinib, an inhibitor of the epidermal growth factor receptor tyrosine kinase, in symptomatic patients with non-small cell lung cancer: a randomized trial JAMA 2003 290 16 2149 2158 10.1001/jama.290.16.2149 14570950

[B12] Mok TS Wu YL Thongprasert S Yang CH Chu DT Saijo N Sunpaweravong P Han B Margono B Ichinose Y Gefitinib or carboplatin-paclitaxel in pulmonary adenocarcinoma N Engl J Med 2009 361 10 947 957 10.1056/NEJMoa0810699 19692680

[B13] Lynch TJ Bell DW Sordella R Gurubhagavatula S Okimoto RA Brannigan BW Harris PL Haserlat SM Supko JG Haluska FG Activating mutations in the epidermal growth factor receptor underlying responsiveness of non-small-cell lung cancer to gefitinib N Engl J Med 2004 350 21 2129 2139 10.1056/NEJMoa040938 15118073

[B14] Paez JG Janne PA Lee JC Tracy S Greulich H Gabriel S Herman P Kaye FJ Lindeman N Boggon TJ EGFR mutations in lung cancer: correlation with clinical response to gefitinib therapy Science 2004 304 5676 1497 1500 10.1126/science.1099314 15118125

[B15] Pao W Miller V Zakowski M Doherty J Politi K Sarkaria I Singh B Heelan R Rusch V Fulton L EGF receptor gene mutations are common in lung cancers from "never smokers" and are associated with sensitivity of tumors to gefitinib and erlotinib Proc Natl Acad Sci U S A 2004 101 36 13306 13311 10.1073/pnas.0405220101 15329413 PMC516528

[B16] Kosaka T Yatabe Y Endoh H Kuwano H Takahashi T Mitsudomi T Mutations of the epidermal growth factor receptor gene in lung cancer: biological and clinical implications Cancer Res 2004 64 24 8919 8923 10.1158/0008-5472.CAN-04-2818 15604253

[B17] Takano T Ohe Y Sakamoto H Tsuta K Matsuno Y Tateishi U Yamamoto S Nokihara H Yamamoto N Sekine I Epidermal growth factor receptor gene mutations and increased copy numbers predict gefitinib sensitivity in patients with recurrent non-small-cell lung cancer J Clin Oncol 2005 23 28 6829 6837 10.1200/JCO.2005.01.0793 15998907

[B18] Rosell R Moran T Queralt C Porta R Cardenal F Camps C Majem M Lopez-Vivanco G Isla D Provencio M Screening for epidermal growth factor receptor mutations in lung cancer N Engl J Med 2009 361 10 958 967 10.1056/NEJMoa0904554 19692684

[B19] Morita S Okamoto I Kobayashi K Yamazaki K Asahina H Inoue A Hagiwara K Sunaga N Yanagitani N Hida T Combined survival analysis of prospective clinical trials of gefitinib for non-small cell lung cancer with EGFR mutations Clin Cancer Res 2009 15 13 4493 4498 10.1158/1078-0432.CCR-09-0391 19531624

[B20] Sequist LV Martins RG Spigel D Grunberg SM Spira A Janne PA Joshi VA McCollum D Evans TL Muzikansky A First-line gefitinib in patients with advanced non-small-cell lung cancer harboring somatic EGFR mutations J Clin Oncol 2008 26 15 2442 2449 10.1200/JCO.2007.14.8494 18458038

[B21] Maemondo M Inoue A Kobayashi K Sugawara S Oizumi S Isobe H Gemma A Harada M Yoshizawa H Kinoshita I Gefitinib or chemotherapy for non-small-cell lung cancer with mutated EGFR N Engl J Med 2010 362 25 2380 2388 10.1056/NEJMoa0909530 20573926

[B22] Mitsudomi T Morita S Yatabe Y Negoro S Okamoto I Tsurutani J Seto T Satouchi M Tada H Hirashima T Gefitinib versus cisplatin plus docetaxel in patients with non-small-cell lung cancer harbouring mutations of the epidermal growth factor receptor (WJTOG3405): an open label, randomised phase 3 trial Lancet Oncol 2010 11 2 121 128 10.1016/S1470-2045(09)70364-X 20022809

[B23] Mitsudomi T Yatabe Y Mutations of the epidermal growth factor receptor gene and related genes as determinants of epidermal growth factor receptor tyrosine kinase inhibitors sensitivity in lung cancer Cancer Sci 2007 98 12 1817 1824 10.1111/j.1349-7006.2007.00607.x 17888036 PMC11159145

[B24] Kobayashi S Boggon TJ Dayaram T Janne PA Kocher O Meyerson M Johnson BE Eck MJ Tenen DG Halmos B EGFR mutation and resistance of non-small-cell lung cancer to gefitinib N Engl J Med 2005 352 8 786 792 10.1056/NEJMoa044238 15728811

[B25] Nagai Y Miyazawa H Huqun ZZ Tanaka T Udagawa K Kato M Fukuyama S Yokote A Kobayashi K Kanazawa M Genetic heterogeneity of the epidermal growth factor receptor in non-small cell lung cancer cell lines revealed by a rapid and sensitive detection system, the peptide nucleic acid-locked nucleic acid PCR clamp Cancer Res 2005 65 16 7276 7282 10.1158/0008-5472.CAN-05-0331 16105816

[B26] Lyamichev V Mast AL Hall JG Prudent JR Kaiser MW Takova T Kwiatkowski RW Sander TJ de Arruda M Arco DA Polymorphism identification and quantitative detection of genomic DNA by invasive cleavage of oligonucleotide probes Nat Biotechnol 1999 17 3 292 296 10.1038/7044 10096299

[B27] Therasse P Arbuck SG Eisenhauer EA Wanders J Kaplan RS Rubinstein L Verweij J Van Glabbeke M van Oosterom AT Christian MC New guidelines to evaluate the response to treatment in solid tumors. European Organization for Research and Treatment of Cancer, National Cancer Institute of the United States, National Cancer Institute of Canada J Natl Cancer Inst 2000 92 3 205 216 10.1093/jnci/92.3.205 10655437

[B28] Davidoff AJ Tang M Seal B Edelman MJ Chemotherapy and survival benefit in elderly patients with advanced non-small-cell lung cancer J Clin Oncol 2010 28 13 2191 2197 10.1200/JCO.2009.25.4052 20351329

[B29] Kudoh S Takeda K Nakagawa K Takada M Katakami N Matsui K Shinkai T Sawa T Goto I Semba H Phase III study of docetaxel compared with vinorelbine in elderly patients with advanced non-small-cell lung cancer: results of the West Japan Thoracic Oncology Group Trial (WJTOG 9904) J Clin Oncol 2006 24 22 3657 3663 10.1200/JCO.2006.06.1044 16877734

[B30] The Elderly Lung Cancer Vinorelbine Italian Study Group Effects of vinorelbine on quality of life and survival of elderly patients with advanced non-small-cell lung cancer J Natl Cancer Inst 1999 91 1 66 72 9890172 10.1093/jnci/91.1.66

[B31] Gridelli C The ELVIS trial: a phase III study of single-agent vinorelbine as first-line treatment in elderly patients with advanced non-small cell lung cancer. Elderly Lung Cancer Vinorelbine Italian Study Oncologist 2001 6 Suppl 1 4 7 11181997 10.1634/theoncologist.6-suppl_1-4

[B32] Gridelli C Perrone F Gallo C Cigolari S Rossi A Piantedosi F Barbera S Ferrau F Piazza E Rosetti F Chemotherapy for elderly patients with advanced non-small-cell lung cancer: the Multicenter Italian Lung Cancer in the Elderly Study (MILES) phase III randomized trial J Natl Cancer Inst 2003 95 5 362 372 10.1093/jnci/95.5.362 12618501

[B33] Quoix EA Oster J Westeel V Pichon E Weekly paclitaxel combined with monthly carboplatin versus single-agent therapy in patients age 70 to 89: IFCT-0501 randomized phase III study in advanced non-small cell lung cancer (NSCLC) J Clin Oncol 2010 2010 suppl; abstr 2 18s

